# Supplementary material for: Human papillomavirus-related psychosocial impact of patients with genital warts in China: a hospital-based cross-sectional study
Source: BMC Public Health. 2014 Jul 21;14:739. doi: 10.1186/1471-2458-14-739 (PMC4223584; doi:10.1186/1471-2458-14-739)
Supplement: Additional file 1 — HPV IMPACT PROFILE. [file 1471-2458-14-739-S1.pdf]

HPV IMPACT PROFILE

We are interested in understanding how women's gynecological health or reproductive health may affect their everyday life. Please think about <u>your most recent</u> experience visiting your gynecologist, having a Pap test, and any other procedures you have had done and test results when answering these questions. Check the <u>ONE</u> best answer for each question.

1. When I think about my recent gynecology exam or test results, I feel good about myself.

Not at all	A little			Somewhat			A great deal			Extremely
☐	☐	☐	☐	☐	☐	☐	☐	☐	☐	☐
0	1	2	3	4	5	6	7	8	9	10

2. When I think about my recent gynecology exam or test results, I feel anxious.

Not at all	A little			Somewhat			A great deal			Extremely
☐	☐	☐	☐	☐	☐	☐	☐	☐	☐	☐
0	1	2	3	4	5	6	7	8	9	10

3. I feel my recent gynecology test results were unexpected.

Not at all	A little			Somewhat			A great deal			Extremely
☐	☐	☐	☐	☐	☐	☐	☐	☐	☐	☐
0	1	2	3	4	5	6	7	8	9	10

4. When I think about my recent gynecology exam or test results, I feel in control of my health.

Not at all	A little			Somewhat			A great deal			Extremely
☐	☐	☐	☐	☐	☐	☐	☐	☐	☐	☐
0	1	2	3	4	5	6	7	8	9	10

5. When I think about my recent gynecology exam or test results, I feel depressed.

Not at all	A little			Somewhat			A great deal			Extremely
☐	☐	☐	☐	☐	☐	☐	☐	☐	☐	☐
0	1	2	3	4	5	6	7	8	9	10

6. After my recent gynecology exam or test results, I feel I can concentrate as well as usual on everyday matters.

Not at all		A little			Somewhat			A great deal		Extremely
0	1	2	3	4	5	6	7	8	9	10

7. When I think about my recent gynecology exam or test results, I feel something is seriously wrong with me.

Not at all		A little			Somewhat			A great deal		Extremely
0	1	2	3	4	5	6	7	8	9	10

8. When I think about my recent gynecology exam or test results, I feel angry.

Not at all		A little			Somewhat			A great deal		Extremely
0	1	2	3	4	5	6	7	8	9	10

9. After my recent gynecology exam or test results, I feel confident my partner will accept me.

Not at all		A little			Somewhat			A great deal		Extremely
0	1	2	3	4	5	6	7	8	9	10

10. When I think about my recent gynecology exam or test results, I feel my body is sexually attractive.

Not at all		A little			Somewhat			A great deal		Extremely
0	1	2	3	4	5	6	7	8	9	10

11. When I think about my recent gynecology exam or test results, I feel ashamed.

Not at all		A little			Somewhat			A great deal		Extremely
0	1	2	3	4	5	6	7	8	9	10

12. I feel concerned about having genital warts.

Not at all		A little			Somewhat			A great deal		Extremely
0	1	2	3	4	5	6	7	8	9	10

13. I am worried there are no treatments to cure genital warts.

Not at all		A little			Somewhat			A great deal		Extremely
0	1	2	3	4	5	6	7	8	9	10

14. After my recent gynecology exam or test results, I feel optimistic about my future gynecological health.

Not at all	A little			Somewhat			A great deal			Extremely
☐ 0	☐ 1	☐ 2	☐ 3	☐ 4	☐ 5	☐ 6	☐ 7	☐ 8	☐ 9	☐ 10

15. I am worried about having abnormal Pap test results.

Not at all	A little			Somewhat			A great deal			Extremely
☐ 0	☐ 1	☐ 2	☐ 3	☐ 4	☐ 5	☐ 6	☐ 7	☐ 8	☐ 9	☐ 10

16. I am worried that there is no cure for what causes an abnormal Pap test.

Not at all	A little			Somewhat			A great deal			Extremely
☐ 0	☐ 1	☐ 2	☐ 3	☐ 4	☐ 5	☐ 6	☐ 7	☐ 8	☐ 9	☐ 10

17. I am worried about my fertility because of my recent gynecological health or test results.

Not at all	A little			Somewhat			A great deal			Extremely
☐ 0	☐ 1	☐ 2	☐ 3	☐ 4	☐ 5	☐ 6	☐ 7	☐ 8	☐ 9	☐ 10

18. I am concerned I will get cervical cancer in the future.

Not at all	A little			Somewhat			A great deal			Extremely
☐ 0	☐ 1	☐ 2	☐ 3	☐ 4	☐ 5	☐ 6	☐ 7	☐ 8	☐ 9	☐ 10

19. I am worried that there are no treatments to cure cervical cancer.

Not at all	A little			Somewhat			A great deal			Extremely
☐ 0	☐ 1	☐ 2	☐ 3	☐ 4	☐ 5	☐ 6	☐ 7	☐ 8	☐ 9	☐ 10

20. I am worried about having pain during future gynecologist visits.

Not at all	A little			Somewhat			A great deal			Extremely
☐ 0	☐ 1	☐ 2	☐ 3	☐ 4	☐ 5	☐ 6	☐ 7	☐ 8	☐ 9	☐ 10

21. After my recent gynecology exam or test results, I am worried that having sex with my partner may give him/her an infection.

Not at all	A little			Somewhat			A great deal			Extremely
☐ 0	☐ 1	☐ 2	☐ 3	☐ 4	☐ 5	☐ 6	☐ 7	☐ 8	☐ 9	☐ 10

22. After my recent gynecology exam or test results, I am worried that having sex with my partner may give _me_ an infection.

Not at all	A little			Somewhat			A great deal			Extremely
0	1	2	3	4	5	6	7	8	9	10

23. I felt disgusted by my recent gynecology exam or test results.

Not at all	A little			Somewhat			A great deal			Extremely
0	1	2	3	4	5	6	7	8	9	10

24. After my recent gynecology exam or test results, I am having less sex.

Not at all	A little			Somewhat			A great deal			Extremely
0	1	2	3	4	5	6	7	8	9	10

25. After my recent gynecological exam or test results, I feel satisfied with my sex life.

Not at all	A little			Somewhat			A great deal			Extremely
0	1	2	3	4	5	6	7	8	9	10

26. After my recent gynecological exam or test results, the quality of my sleep has decreased.

Not at all	A little			Somewhat			A great deal			Extremely
0	1	2	3	4	5	6	7	8	9	10

27. I felt relaxed after my recent gynecological exam.

Not at all	A little			Somewhat			A great deal			Extremely
0	1	2	3	4	5	6	7	8	9	10

28. I felt my recent gynecology procedures were embarrasing.

Not at all	A little			Somewhat			A great deal			Extremely
0	1	2	3	4	5	6	7	8	9	10

29. I felt the medical procedures at my recent gynecological exam were uncomfortable.

Not at all	A little			Somewhat			A great deal			Extremely
0	1	2	3	4	5	6	7	8	9	10
